# Non-convex optimization for inverse problem solving in computer-generated holography

**DOI:** 10.1038/s41377-024-01446-w

**Published:** 2024-07-09

**Authors:** Xiaomeng Sui, Zehao He, Daping Chu, Liangcai Cao

**Affiliations:** 1 Department of Precision Instruments, Tsinghua University, Beijing, 100084 China; 2 Department of Engineering, Centre for Photonic Devices and Sensors, University of Cambridge, 9 JJ Thomson Avenue, Cambridge, CB3 0FA UK; 3 Cambridge University Nanjing Centre of Technology and Innovation, 23 Rongyue Road, Jiangbei New Area, Nanjing, 210000 China

**Keywords:** Displays, Imaging and sensing

## Abstract

Computer-generated holography is a promising technique that modulates user-defined wavefronts with digital holograms. Computing appropriate holograms with faithful reconstructions is not only a problem closely related to the fundamental basis of holography but also a long-standing challenge for researchers in general fields of optics. Finding the exact solution of a desired hologram to reconstruct an accurate target object constitutes an ill-posed inverse problem. The general practice of single-diffraction computation for synthesizing holograms can only provide an approximate answer, which is subject to limitations in numerical implementation. Various non-convex optimization algorithms are thus designed to seek an optimal solution by introducing different constraints, frameworks, and initializations. Herein, we overview the optimization algorithms applied to computer-generated holography, incorporating principles of hologram synthesis based on alternative projections and gradient descent methods. This is aimed to provide an underlying basis for optimized hologram generation, as well as insights into the cutting-edge developments of this rapidly evolving field for potential applications in virtual reality, augmented reality, head-up display, data encryption, laser fabrication, and metasurface design.

## Introduction

Holography is a long-existing concept first raised by Dennis Gabor in the late 1940s, which aimed at improving resolution in electron microscopy^1^. In the 1960s, the development of laser technology enabled practical optical holography^2,3^. Early demonstration of optical holography can be described with two steps: interferential recording of an object wavefront and diffractive reconstruction from a hologram. Recent advancements in digital devices have enabled both the recording and the reconstruction processes to be performed computationally. One branch of holography involves optically recording an object wavefront and computationally reconstructing it from a digital hologram^4,5^, commonly referred to as digital holography^6^. This approach enables promising applications such as imaging, measurement, and detection. Another branch of holography involves computationally generating a hologram and optically reconstructing an object’s wavefront, commonly referred to as computer-generated holography (CGH), which provides an approach to digitally modulate a volumetric wavefront^7^. This technology, half inherited from optical holography and half advanced by computer science, has become an emerging focus of academia and industry^8–10^.

**Fig. 1:**
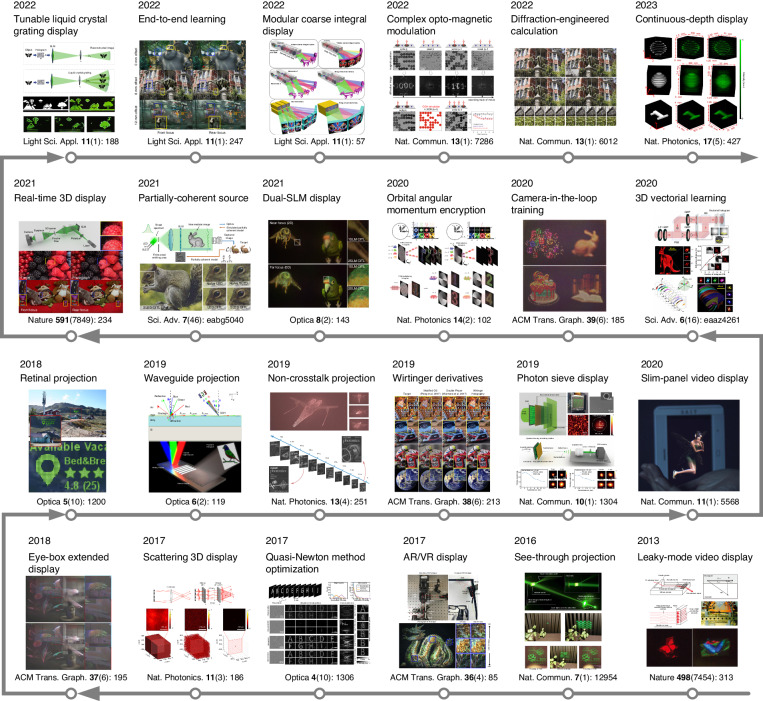
Recent breakthroughs in CGH enable the development of 3D holographic display^17,18,25–27,40,49,51,52,61,66,69,133,141,208,213^ and AR/VR display^33,39,41,50,132^. With the extended capability of CGH, new thoughts are brought into interdisciplinary investigations such as optogenetics^68^ and optical encryption^20,215^. References^17,18,20,27,49,208^ are reprinted with permission from Springer Nature. References^39,50,68,141^ are reprinted with permission from © Optical Society of America. References^33,40,132,133^ are reprinted with permission from © ACM. Reference^215^ is reprinted with permission from © AAAS. References^25,26,41,51,52,61,66,69,213^ are reprinted under a Creative Commons Attribution 4.0 International License

Computer-generated holograms, encoded on various types of holographic media, enable a wide range of applications. Holograms fabricated as diffractive optical elements (DOEs)^11^ or metasurfaces^12–14^ can reproduce specific spatial light fields, achieving structured light projection^15–17^, data storage^18,19^, and optical encryption^20–24^. With refreshable devices like spatial light modulators (SLMs)^25–27^, as is shown in Fig. 1, CGH is able to assist many fields of investigations, including three-dimensional display, holographic lithography^28^, optical trapping^29^, and optogenetics^30–32^. In recent years, CGH also boosts the birth and growth of potential markets of virtual reality (VR)^33–38^, augmented reality (AR)^39–42^, head-up display^43–45^, holographic printing^46^, optical communication^47^, and optical computing^48^. Although these applications and fields of investigation involve the encoding of holograms with various elements^49,50^ and devices^51,52^, the algorithms for hologram synthesis are similar and can be universally applied^53^. Therefore, computing a hologram for faithful reconstructions is an issue closely linked to fundamental physics and is widely investigated in general fields of optics. The computation of holograms is a strict adherence to the physics basis of holography while being a compromise to the existing hardware^54^, which requires holograms to be phase-only, amplitude-only, or rarely complex-amplitude. The object wave used for CGH can be reconstructed from either an interference pattern with a reference beam^55,56^ or a diffraction pattern of the object^57^. In early investigations^58^, holograms computed from diffractive wave propagations were found to be more energy-efficient compared to those computed from interferences. These wave-propagation methods yielded complex holograms (CHs) using computer-guided plotters employing the detour phase principle^59^. Subsequently, phase-only holograms (POHs) were proposed^60^, generated on the assumption that a scattered wavefront can be reconstructed with only the phase term. Proper computation of a diffraction-based hologram is essential for the optical reconstruction of CHs and POHs, and it gradually becomes vital for generating amplitude-only holograms^61^.

**Fig. 2:**
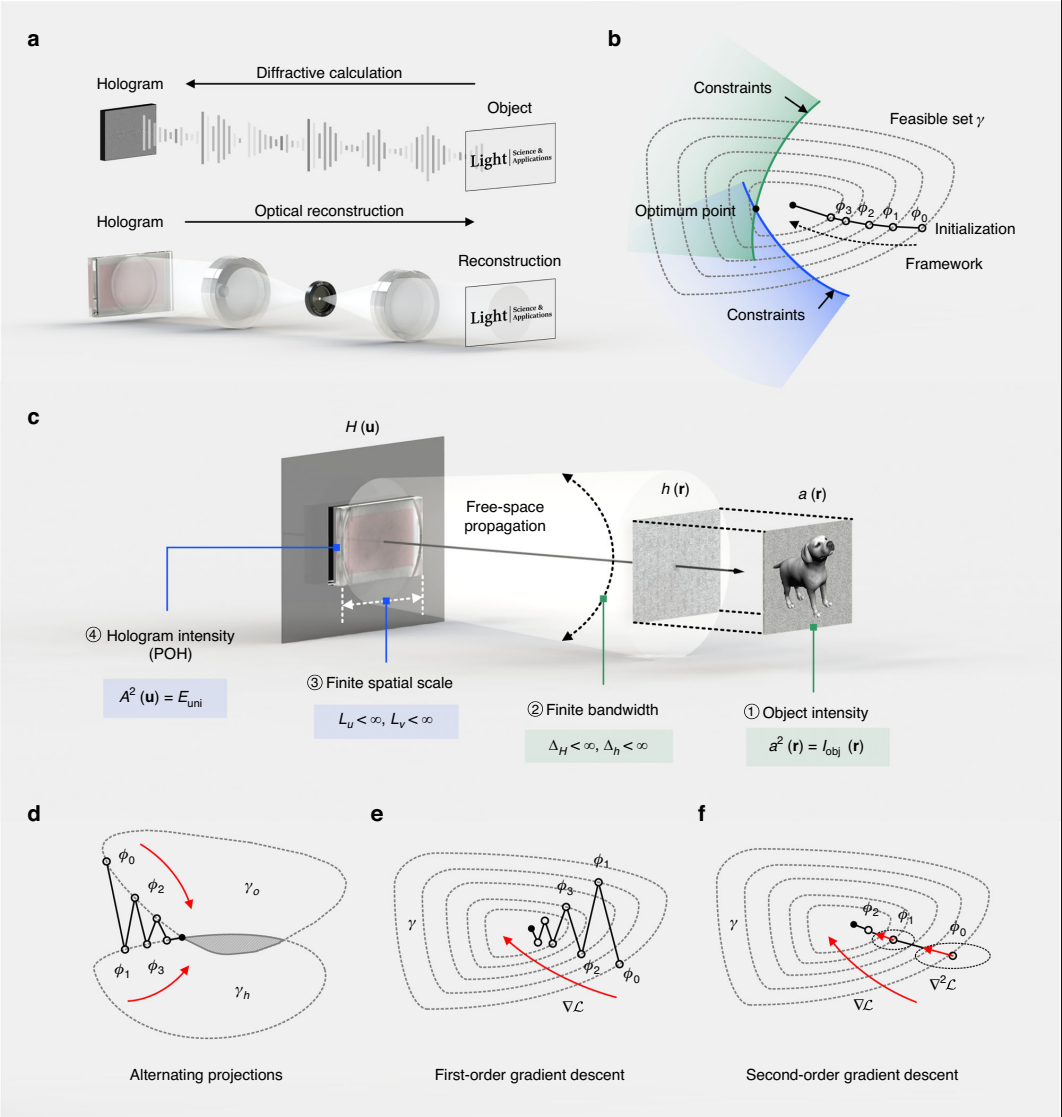
The inverse problem existing in computer-generated holography (CGH) and fundamental concerns of optimization. **a** The synthesis of computer-generated holograms can be described as an inverse problem. **b** Constraints, frameworks, and the initialization condition need to be considered in hologram optimization. **c** Constraints are imposed by physics and hardware in hologram optimization. Some optimization frameworks are widely used in hologram optimization: **d** alternating projections, where elementary projections occur to approach the intersection points of two or more enclosed feasible sets; **e** first-order gradient descent methods, where a local optimal solution is searched along the gradient descent direction; **f** second-order gradient descent methods, where the “steepest” gradient descent direction is further found with the assistance of second-order gradient

The fundamental concern of the diffraction-based hologram computation can be neatly described as solving a hologram from a given intensity distribution of the object^62^, as is shown in Fig. 2a, which is an inverse problem with constraints imposed by physical basis and hardware implementation. This inverse problem is relatively different from the phase retrieval problems in imaging because a hologram satisfying all the constraints and reconstructing an artificial intensity distribution is not ensured to exist^63^. Nevertheless, the relation between the reconstructed wavefront and its intensity is ill-conditioned^64^. Multiple candidate holograms that approximately satisfy the constraints can be solved through the non-convex optimization^65^. Various optimization algorithms are thus introduced in hologram synthesis and rapidly bring about breakthroughs for CGH in noise reduction^66^, contrast enhancement^67^, crosstalk suppression^68^, and computing acceleration^69^. With the significant enhancement of CGH capabilities through optimization algorithms, achieving appropriate hologram optimization tailored to the physics process emerges as a central target, which ensures desired optical reconstructions. The motivation of computing all kinds of holograms with faithful reconstructions has pushed forward the advancement of optimization algorithms for CGH.

This review article focuses on optimization algorithms for hologram generation in CGH, which have become essential tools in hologram synthesis over the past decades. Concluded from existing optimization algorithms appropriately adapted to CGH inverse problem solving, as is shown in Fig. 2b, some crucial factors need to be considered, including constraints, framework, and initialization. Constraints refer to the conditions that a candidate solution should satisfy, usually modeled with the physical basis and the hardware implementation of CGH. Restricted by the constraints, possible solutions of the inverse problem constitute an enclosed feasible set *γ*, within which the optimization algorithms search for a solution locally or globally. The optimization framework chosen determines the path of searching within the feasible set, leading to variations in computing time, storage, and sometimes even convergence points. Initialization, especially the choice of the initialization condition, has a significant impact on the final convergence point. With a comprehensive overview of the original and recent designs of optimization algorithms, suitable handling of core issues concerning feasible hologram optimization is illustrated, offering a basis for applying optimized holograms to various fields of investigation.

## Constraints

Optimization algorithms are designed to retrieve a computer-generated hologram $H({\bf{u}})=A({\bf{u}}){e}^{i\phi ({\bf{u}})}$ from a specific object wave $h({\bf{r}})=a({\bf{r}}){e}^{i\varphi ({\bf{r}})}$ in an inverse problem solving situation of CGH, where *a*(**r**) is the amplitude, *φ*(**r**) is the phase, ${\bf{r}}=(x,y)$ and ${\bf{u}}=(u,v)$ are the position vectors on the object plane and the hologram plane, respectively. As shown in Fig. 2c, such an optimization is restricted by several constraints^64,65^:

① Object intensity $${a}^{2}({\bf{r}})={I}_{{\rm{obj}}}({\bf{r}})$$;
② Finite bandwidth ${\Delta }_{H} \,<\, \infty$ and ${\Delta }_{h} \,<\, \infty$;
③ Finite spatial scale ${L}_{u} \,<\, \infty$ and ${L}_{v} \,<\, \infty$;
④ Hologram intensity for POHs ${A}^{2}({\bf{u}})={E}_{{\rm{uni}}}$.

Constraint ① describes the pre-defined object intensity, which is fed as a constraint in optimization to ensure a faithful reconstruction. Constraint ② describes a finite bandwidth throughout the process of propagation, where Δ_*H*_ and Δ_*h*_ represent the hologram and object wave bandwidths, respectively. Constraint ③ describes the finite spatial scale of the hologram, which is usually conducted by zero padding in practical computation. *L*_*u*_ and *L*_*v*_ represent the length and the width of the hologram to be optimized respectively. Constraint ④ is optional and specially imposed due to a constant hologram intensity of POHs, where ${E}_{{\rm{uni}}}$ is the hologram intensity.

These constraints ensure a correct optimization adapting to the physical model of holography and enable a faithful reveal of the reconstruction performance in numerical simulations. Errors and omissions of the constraints imposed on optimization can lead to a mismatch between the numerical and optical reconstructions^70^, usually shown as obviously excessive speckle noise and artifacts appearing in the optical reconstructions compared to the numerical ones^71^. Given all these constraints imposed, we can deduce that the object phase *φ*(**r**) is the only unsettled input factor in optimization, which provides a degree of freedom to design the hologram^72^. With different initializations of *φ*(**r**), the path and the outcome of optimization can differ greatly, leading to various performances of optical reconstructions.

The effectiveness of optimization is largely decided by whether the constraints are correctly imposed or not. The way the constraints are imposed is determined not only by the type of holograms but also by the diffractive calculation method utilized to implement computational free-space propagation. Existing diffractive calculation methods demonstrated for hologram optimization are mainly conducted by parallel computation based on the Fourier transform^73^. Among them, using a single fast Fourier Transform (FFT) to compute Fraunhofer diffraction at infinity is one of the mostly used propagation strategies^74^, which is easy and simple enough to implement in research. Fresnel diffraction is often chosen to generate far-field holograms^75^, with diffractive calculation methods including the single Fourier-transform Fresnel (SFT-FR) approach^76^, the Fresnel transfer function approach, and the Fresnel impulse response approach^77^. Besides, some methods derived directly from the first Rayleigh-Sommerfeld integral theory present high practicability in CGH^78^, covering both near-field and far-field propagations, among which the representative examples are the Rayleigh-Sommerfeld convolution method^79^ and its corresponding Fourier form angular spectrum method (ASM)^80^.

Here, we uniformly utilize the single FFT method^81,82^ to illustrate 2D hologram optimization algorithms and the band-limited ASM^83^ for the 3D case. Given different diffractive calculation methods, the most controversial part of imposing constraints lies in defining a proper bandwidth restriction to describe constraint ②, which is discussed in the following sections.

## Framework

The optimization algorithm searches for an optimal solution as the desired hologram within a closed set restricted by the constraints. Several effective optimization frameworks are widely used, including alternating projection methods, which are depicted in Fig. 2d, and gradient descent methods. More specifically, the gradient descent methods also incorporate the first-order gradient descent as depicted in Fig. 2e, and the second-order gradient descent as depicted in Fig. 2f, of which the representative examples are stochastic gradient descent (SGD) and the quasi-Newton method respectively. Some other optimization frameworks are also applied to CGH, like iterative shrinkage-thresholding algorithms^84^ and simulated annealing^85^. Yet, these frameworks are less widely used in hologram synthesis.

### Alternating projections

**Fig. 3:**
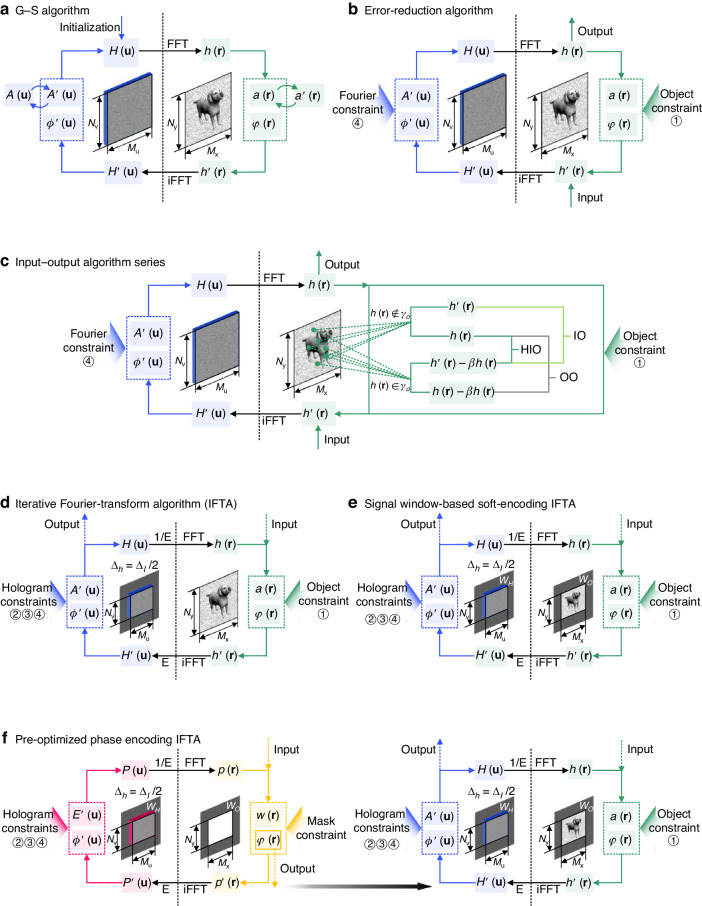
Schemes of alternating projection algorithms applied in phase retrieval or hologram optimization. **a** Gerchberg-Saxton algorithm (G-S algorithm)^88^. **b** Error reduction algorithm^89^. **c** Input-output algorithm series, including the basic input-output algorithm (IO), the output-output algorithm (OO), and the hybrid input-output algorithm (HIO)^90^. **d** Iterative Fourier-transform algorithm (IFTA)^70^. **e** Signal window based soft-encoding IFTA^71^. **f** Phase mask based on IFTA^106^

The alternating projections describe a category of optimization methods to approximate a point in the intersection of two or more enclosed feasible sets separately restricted by different constraints^86^. This is achieved by a pair of elementary projections repeatedly occurring in the optimization, which construct an iterative computation loop^87^. The Gerchberg-Saxton algorithm (G-S algorithm)^88^ was published in 1972 as the first account of alternating projections solving a phase retrieval problem, in which a pair of projections were used to reconstruct phase from two intensity measurements. As is shown in Fig. 3a, the phase of a specific wavefront is retrieved with the amplitudes on both the object plane $a^{\prime} ({\bf{r}})$ and the Fourier plane $A^{\prime} ({\bf{u}})$ repeatly replaced by known distributions, which are discretized into a ${M}_{x}\times {N}_{y}$ matrix and a ${M}_{u}\times {N}_{v}$ matrix respectively. Later presented by Fienup, this alternating scheme was improved to solve the problem of finding a Fourier transform pair satisfying the constraints in both planes, which was known as the error reduction algorithm^89^. As is shown in Fig. 3b, the error reduction algorithm enhances the flexibility of the alternating projections and enables various non-negative constraints to be imposed on dual planes. After that, a series of input-output algorithms^90^ were further developed to accelerate the convergence of the error reduction algorithm. Input-output algorithms differ from error reduction algorithms during the nonlinear operation on the object plane. In the basic input-output (IO) algorithm, the object constraints are imposed only on the points that violate the object constraints $h({\bf{r}})\notin {\gamma }_{o}$. Such a change is based on the idea that the distribution of $h^{\prime} ({\bf{r}})$ for the next iteration does not have to satisfy the object constraints but is given to drive a desired and constrained output. Based on this thought, different nonlinear operations can be introduced on the object plane, as is shown in Fig. 3c, through which the output-output (OO) algorithm and the hybrid input-output (HIO) algorithm were developed. The HIO algorithm possesses an advantage in avoiding the stagnation problem, which occurs sometimes in the OO algorithm and prevents the optimization from getting close to the solution. In some situations, the unique characteristics of the HIO algorithm set it apart from the input-output algorithm series for its imaging applications^91,92^. All these alternating projection algorithms are now widely studied as early solutions to phase retrieval problems. They have been derived into different forms for specific applications^93^.

Concurrent with the advancement of algorithm flows, numerous efforts are made to adapt alternating projection algorithms into the physical model of CGH. Specially in hologram optimizations, alternating projections are applied to two enclosed sets associated with potential object solutions *γ*_*o*_ and potential hologram solutions *γ*_*h*_, which are restricted by object constraints and hologram constraints respectively. The preliminary applications of alternating projections to solve the hologram synthesis problem in CGH appeared just before the first report of the G-S algorithm. Gabel and Liu proposed a flow chart with Fourier transform pairs to minimize binary hologram reconstruction errors in 1970^94^. Hirsch et al.^95^ invented a technique for optimizing Kinoforms with a loop of forward and backward propagations in 1971. In the early studies on optimization algorithms for CGH^96^, Fourier transform pairs were widely used for diffractive calculation since they could easily simulate Fraunhofer diffractions at infinity. When this scheme became known as the error-reduction algorithm and was extended into an input-output algorithm series, Fienup clarified that such an iterative Fourier transform loop is the alternating projections to solve a hologram from specific constraints on the object plane and hologram plane, rather than simple exchanges of amplitude values on dual planes^62,97^.

With the extension of the categories of constraints, holograms can be computed well in line with the theoretical model of holography. In 1988, an iterative Fourier transform algorithm (IFTA) specially applied to CGH was proposed by Wyrowski and Bryngdahl^70^. As is shown in Fig. 3d, the IFTA scheme additionally includes the bandwidth constraint ② and the spatial scale constraint ③ in computation. A dense sampling on the object plane was proposed by applying the bandwidth constraint of ${\Delta }_{h}={\Delta }_{I}/2$, where Δ_*I*_ was the bandwidth of the object intensity field^79^. The theoretical basis of this operation is that the spectra of the object wave and the spectra of the object intensity are related by autocorrelation. Minor errors and noises were able to be revealed in the numerical reconstruction, as a joint result of physical constraints and dense sampling. The performance of an optimized hologram can be sufficiently improved in optical reconstruction, with a desired object intensity fed as a constraint on the object plane. In the subsequent studies on IFTA, signal windows, energy operators, and soft encoding are introduced into the algorithm. The signal window utilizes the redundancy of hologram design and distributes the noises outside the signal window^98^. Because the object and the hologram are both finite in spatial scale, the object wave is defined within a window *W*_*O*_ in computation, and the corresponding hologram is restricted within a window *W*_*H*_, as shown in Fig. 3e. On the object plane, a weighting factor is usually introduced to adjust the energy distribution ratio in and out of the window *W*_*O*_^71^, so that the object intensity constraint is only imposed within the signal window. On the hologram plane, with *W*_*H*_ introduced as the bandwidth and special scale constraint on the hologram. The definition of the signal window allows for the use of amplitude freedom other than phase freedom on the object plane while optimizing holograms^99,100^. Introducing the double freedoms loosens the object intensity constraint ①, which exchanges for higher reconstructing accuracy by allowing noises in less-concerned areas^101,102^. Coordinated with quadratic phase initializations, IFTA algorithms with double freedoms can achieve speckle-free reconstructions^103,104^. The introduction of signal windows makes use of the dark area outside the object window to accommodate minor errors produced in the optimization of a POH, which also accelerates the process of reaching to the convergence. To ensure an appropriate distribution of the signal and the noise, energy operators *E* and 1/*E* are both used to normalize the intensities in the transformation between the object plane and the hologram plane. The soft encoding strategy was introduced to control the direction of optimization in IFTA. One way of achieving soft encoding is to apply a varying operation on the hologram plane^71^. This varying operation leads to minimal changes in the object phase and results in a smooth transition towards the desired optimizing direction. The signal window and the soft encoding, when combined, work for various types of initial object phases in IFTA, offering this algorithm a great deal of flexibility. Apart from the random phase, which was originally used as the object phase for Kinoform, some remarkable attempts at various phase initialization also include the quadratic phase^71^, the constant phase^105^, and the pre-optimized phase^64^. The utilization of constant phase and quadratic phase is driven by a demand for speckle suppression in CGH. The pre-optimized phase is utilized to develop a less iterative^106,107^ or noniterative^108–110^ approach to quickly generate holograms, which greatly accelerates the computation. As is shown in Fig. 3f, such algorithms can be divided into two steps: the first step optimizes a random phase mask within the signal window, which to the largest extent satisfies the constraints on the dual planes; the second step optimizes the hologram with the pre-optimized phase input as the initial object phase. The pre-optimized phase can be utilized to initialize object waves with different intensity patterns. And the computation can thus be simplified into a loop of optimizing holograms with only several iterations, or even without iterations.

Despite the introduction of alternating projection algorithms, the reconstruction of CGH can still be plagued by various unwanted effects, including speckle noise, artifacts, and stripes. These effects may trap the optimization into stagnation and remain in reconstruction even after convergence. Thus, some modifications applied to optimization algorithms are also studied. Since the complex distributions on both the object plane and the hologram plane are calculated in iterations, the framework of the alternating projections possesses the flexibility to include manipulations on the object phase^111^, Gaussian illumination^112^, weighting factors^113^, internal processing in the loop^114^, partitioned optimization^115,116^ and multi-loop optimization^117^. With highly promoted reconstructing accuracy and computation stability enabled by accumulated improvements on alternating projection algorithms, alternating projection algorithms occupy an indispensable part in optimizations of CGH, especially for the synthesis of holograms encoded on static holographic media, like DOEs^118,119^ and metasurfaces^120–122^.

The highly developed schemes enable alternating projection algorithms to be applied to hologram generation based on neural networks in the early stages^123^, which provide training datasets of ground-truth holograms. Despite the quick advancements of CGH combined with various neural networks in recent years^124,125^, a pre-optimization carried out by the alternating projections is still a widely adopted choice for supervised learning^126–129^.

### Stochastic Gradient Descent (SGD)

Because of the existence of specific object intensity distributions, the inverse problem of hologram synthesis in CGH can also be cast as the optimization of a parameterized objective function requiring minimization with respect to its parameters. Since the choice of the objective function is often stochastic and differentiable with respect to its parameters, SGD is considered as an efficient and effective first-order gradient descent ($\nabla {\mathcal{L}}$) framework for optimization. Although such a scheme searching for a local optimal solution along the gradient descent direction has been early applied to phase retrieval^130,131^, SGD was hardly applied to CGH till recent years. In 2019, Chakravarthula et al. chose such a framework to demonstrate the loss functions they introduced for Wirtinger holography^132^. In 2020, Peng et al. utilized SGD to carry out a camera-in-the-loop optimization^133^.

Feasible gradient descent requires the gradient of the object function to be comparable, or in other words, the gradient with respect to the variable is a real value. Yet, a scalar wavefront is complex, which creates a major difficulty in applying gradient descent strategies in hologram optimization. Since POHs only concern the phase component of the holographic wavefront on the hologram plane, the optimization of POHs becomes relatively easy for implementation. With this regard, a POH can be synthesized by constructing an objective function ${ {\mathcal{L}}}_{P}$ associated with a forward model and solving the minimization problem:

1 $${\phi }_{{\rm{opt}}}=\mathop{{\rm{argmin}}}\limits_{\phi }{ {\mathcal{L}}}_{P}[I(\phi ),{I}_{{\rm{obj}}}]$$

**Fig. 4:**
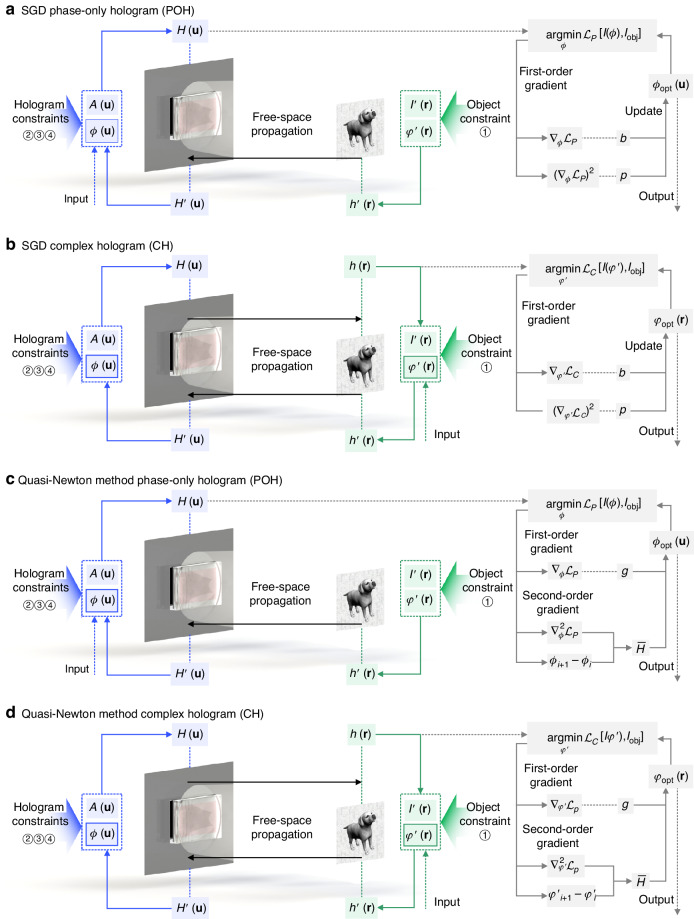
Schemes of the stochastic gradient descent (SGD) method and the quasi-Newton method to optimize holograms. **a** SGD optimization of a phase-only hologram **(**POH). The Adam algorithm updates the POH by calculating the 1st-moment vector *b* and the 2nd-moment vector *p*. **b** SGD optimization of a complex hologram (CH). The Adam algorithm updates the object phase of the CH. **c** Quasi-Newton optimization of a POH. The L-BFGS algorithm updates the POH by calculating the first-order gradient *g* and the inverse Hessian matrix $\bar{H}$. **d** Quasi-Newton optimization of a CH. The L-BFGS algorithm updates the object phase of the CH

${ {\mathcal{L}}}_{P}$ is also referred as a loss function, which usually describes the difference between the reconstructed intensity *I*(*ϕ*) and the object intensity *I*_obj_. *I*(*ϕ*) represents the reconstructed intensity as a function with respect to the POH *ϕ*. Since the reconstructed intensity *I*(*ϕ*) and reconstructed wave *h*(*ϕ*) are linked by an ill-conditioned relation $I(\phi )={|h(\phi )|}^{2}$, the optimization for this hologram synthesis problem is non-convex. In this case, SGD is a relatively efficient optimization method to solve this problem because only first-order partial derivatives concerning all the variables are computed, which have the same computational complexity as just evaluating the objective function. Adam algorithm^134^, shown in Fig. 4a, is one of the most widely implemented SGD-based algorithms for solving the hologram synthesis problem.

**Fig. 5:**
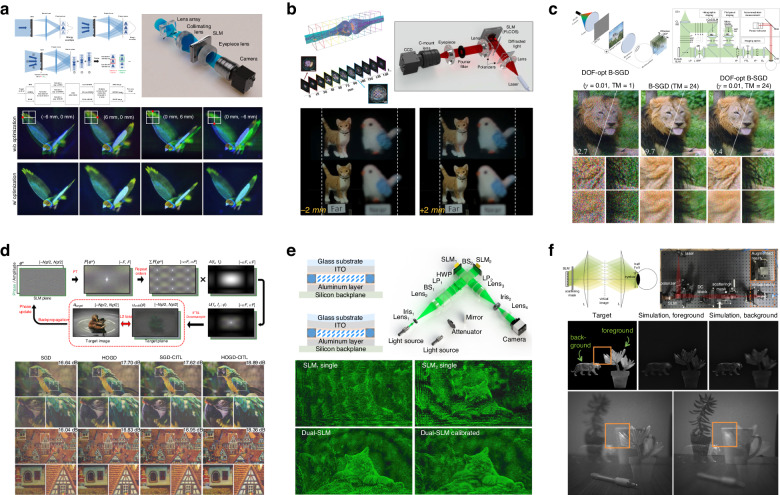
Functional holographic implementations of gradient descent algorithms. **a** Energy envelope expansion^139^. **b** Speckle suppression and contrast enhancement^67^. **c** Improvement in accommodation response^138^. **d** Optimization of high diffraction orders^140^. **e** Uneven liquid crystal modulation^143^. **f** Étendue expansion^84^. References^140,143^ are reprinted with permission from © Optical Society of America. Reference^138^ is reprinted with permission from © ACM. References^67,84,139^ are reprinted under a Creative Commons Attribution 4.0 International License

To minimize the expected value of ${ {\mathcal{L}}}_{P}$, the Adam algorithm updates the exponential moving averages of the partial derivative ${\nabla }_{\phi }{ {\mathcal{L}}}_{P}$ and the elementwise square gradient ${({\nabla }_{\phi }{ {\mathcal{L}}}_{P})}^{2}$, which are described as the 1st-moment vector *b* and the 2nd-moment vector *p*. The optimization only requires the first-order gradient of the objective function ${ {\mathcal{L}}}_{P}$ with respect to the POH *ϕ*, for which reason efficient computation can be achieved with small memory. In such a framework, the object constraint is imposed through the minimization problem itself, while the hologram constraints are applied using the partial derivative ${\nabla }_{\phi }{ {\mathcal{L}}}_{P}$, which is calculated in each update of optimization. Although we write ${ {\mathcal{L}}}_{P}$ in a general form of stochastic scalar function with respect to the reconstructed intensity *I*(*ϕ*) and the object intensity *I*_obj_, the choice of ${ {\mathcal{L}}}_{P}$ is actually of great diversity for hologram synthesis^135,136^. In many CGH implementations, ${ {\mathcal{L}}}_{P}$ is composed of a sum of subfunctions evaluating reconstructing errors^137^, and a normalization term is occasionally added to balance other reconstruction parameters^132,138^. This flexibility allows SGD to achieve a number of functional holographic display demonstrations, as is shown in Fig. 5.

For the synthesis of CHs, some actions need to be taken to ensure the calculated gradient is a real value. Here we accordingly provide an extended scheme for the optimization of CHs using SGD. This is achieved simply by switching the output onto the object plane instead of the hologram plane, given that the object phase is the only floating parameter of the optimization model. The minimization problem is accordingly changed, which can be given as:

2 $${\varphi }_{{\rm{opt}}}=\mathop{{\rm{argmin}}}\limits_{\varphi {\prime} }{ {\mathcal{L}}}_{C}[I(\varphi ^{\prime} ),{I}_{{\rm{obj}}}]$$

where ${ {\mathcal{L}}}_{C}$ is the loss function, and $I(\varphi ^{\prime} )$ is the reconstructed intensity function with respect to the object phase $\varphi ^{\prime}$. Equation (2) determines that the output of this optimization is the object phase, as shown in Fig. 4b, which is again turned into an optimization on a phase distribution. The basis of this scheme is the fact that with a given propagation model and a given object intensity, the complex hologram is only decided by the object phase. Different from that of POHs, the optimization of CHs includes both forward and backward propagations, resulting in a different expression of the gradient ${\nabla }_{\varphi ^{\prime} }{ {\mathcal{L}}}_{C}$. With the optimized object phase ${\varphi }_{{\rm{opt}}}$ and the known object intensity *I*_obj_, the desired CH can be further calculated through an additional diffractive propagation.

Through the combination of a well-performed physical model, a well-chosen initial object phase, and a well-defined loss function, SGD itself has been verified to achieve good reconstructions of POHs, which leads to impressive accomplishments in 3D display with less or without speckles^67,139^. And, because the early adoption of SGD in CGH was linked with camera-in-the-loop optimization^133^, hardware involution was developed in tandem with the promotion of SGD-based hologram synthesis, in which the pattern captured by the camera replaced the reconstructed intensity in optimizations. With camera-in-the-loop optimization, subsequent reconstructions of SGD were further improved significantly by suppressing noises in optical setups, including high diffraction orders^140^, DC term^141^, speckles^66,142^, phase uniformity^143^, and aberrations^144^.

**Fig. 6:**
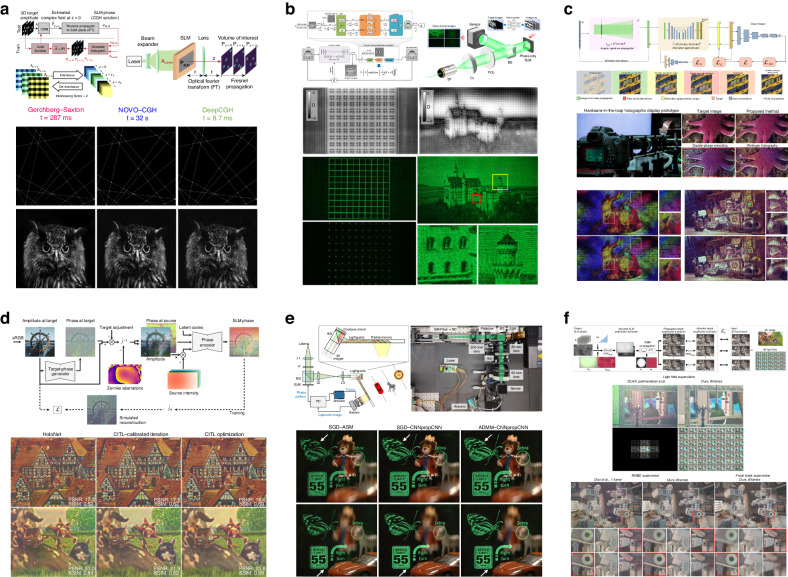
Holographic implementations of neural networks based on gradient descent optimizers. **a** DeepCGH^149^. **b** Holo-encoder^148^. **c** Learned hardware-in-the-loop phase retrieval^151^. **d** Neural holography with camera-in-the-loop training^133^. **e** Neural 3D holography for AR/VR display^152^. **f** Time-multiplexed neural holography^153^. References^148,149^ are reprinted with permission from © Optical Society of America. References^133,151,152^ are reprinted with permission from © ACM. Reference^153^ is reprinted with permission from the authors

Because gradient descent contributes one of the most common optimizers to unsupervised learning of neural networks, the introduction of SGD neatly builds up a connection between CGH and unsupervised learning^145–147^. As the investigations in Fig. 6 have presented, unsupervised neural networks based on SGD accelerate the high-accuracy generation of holograms to ~0.01 s when trained by comparing *I* and *I*_obj_ through a defined loss function rather than large datasets^148–150^. As a step already taken further, unsupervised learning coordinated camera-in-the-loop training perfectly solves the problem of the long processing time that comes with camera-in-the-loop optimization^133^. This scheme pushes the boundaries of optimization-based hologram synthesis by demonstrating a trade-off between computation time, reconstruction quality, and system integration^151–154^.

It is worth noting that the proposal of hologram optimization based on the alternating direction method of multipliers (ADMM) is closely associated with SGD. The ADMM breaks the optimization problem into subproblems with respect to multiple variables, which is achieved by modifying the minimization formula into an augmented Lagrangian form^155,156^. The final hologram is acquired by solving subproblems alternatingly^157^, which requires an inner loop of optimization in the hologram synthesis. The existing scheme of this loop is achieved by the Adam algorithm^152^, which incorporates the diffractive propagation model implemented by matrix manipulation.

### Quasi-Newton method

The quasi-Newton method has become a widely used optimization framework for hologram synthesis in recent years. The quasi-Newton method is a second-order gradient descent method, which minimizes the loss function by constructing and storing a series of matrices that approximate the Hessian or inverse Hessian matrix of the loss function^158^. This operation is associated with the calculation and storage of the second-order gradient (${\nabla }^{2} {\mathcal{L}}$) of the loss function, which requires a larger amount of computation and storage compared with SGD. However, the calculation of the second-order gradient allows for a further search on the optimal direction, providing a possible “steepest” path on gradient descent. The combination of CGH and the quasi-Newton method was introduced by Zhang et al. when they proposed the non-convex optimization for volumetric CGH (NOVO-CGH)^68^. Later in 2019, the quasi-Newton method was also used in Wirtinger holography^132^.

The limited memory BFGS (L-BFGS) algorithm occupies the mainstream in the existing implementation of the quasi-Newton method in hologram synthesis^159,160^, which is chosen for its relatively high efficiency using a limited amount of storage. As depicted in Fig. 4c, ${ {\mathcal{L}}}_{P}$ is the loss function to be minimized given in Eq. (1) for POH synthesis. Similar to SGD, constraint ① in the quasi-Newton method is also imposed through the minimization problem itself, while constraints ②, ③, and ④ are imposed in the expression of the partial derivative ${\nabla }_{\phi }{ {\mathcal{L}}}_{P}$. The POH is updated by calculating both the first-order gradient $g={\nabla }_{\phi }{ {\mathcal{L}}}_{P}$ and the inverse Hessian matrix $\bar{H}$. The inverse Hessian is related to the second-order gradient ${g}_{i+1}-{g}_{i}$, which is estimated by adding up a series of correction matrices of each update in computation^161^. The accumulation of correction matrices is the major reason why the second-order gradient descent requires a greater amount of computation and storage. As a practical approach to implementing the quasi-Newton method, the L-BFGS algorithm has an upper limit for the number of correction matrices that can be stored. Once the number of updates reaches the storage limit, the stored correction matrices are discarded, and the correction matrices are reaccumulated.

Similarly, the quasi-Newton method is able to synthesize CHs by switching the output to the object phase *φ* and calculating the corresponding hologram through diffractive propagation, as is illustrated in Fig. 4d. The minimization problem can also be described with the expression in Eq. (2).

Although the quasi-Newton method and SGD are the second-order gradient descent and first-order gradient descent methods, respectively, they share the same derivation for ${\nabla }_{\phi }{ {\mathcal{L}}}_{P}$ and ${\nabla }_{\varphi ^{\prime} }{ {\mathcal{L}}}_{C}$. Since a second-order gradient is introduced, the quasi-Newton method usually presents higher reliability in searching for the direction of gradient descent, which enables generally higher reconstructing accuracy of holograms with diverse propagation models in practice^68^. However, as a consequence of the large amount of computation in optimization, the quasi-Newton method requires much longer computing time and larger memory compared with other optimization frameworks^149,162^.

### Optimization pipelines for 3D holography

In this section, we describe the way how various optimization frameworks are applied to 3D holography. The flexibility of the optimization frameworks also brings with it a diversity of pipelines for the hologram synthesis of 3D objects. Many diffractive propagation models are applicable for volumetric optimization. Here, we unify them into the band-limited ASM^83^, for a better illustration of different optimization pipelines. Three major existing optimization pipelines for 3D holography are discussed in detail. These pipelines were proposed alongside the advancement of alternating projection algorithms and have been combined with other optimization frameworks.

**Fig. 7:**
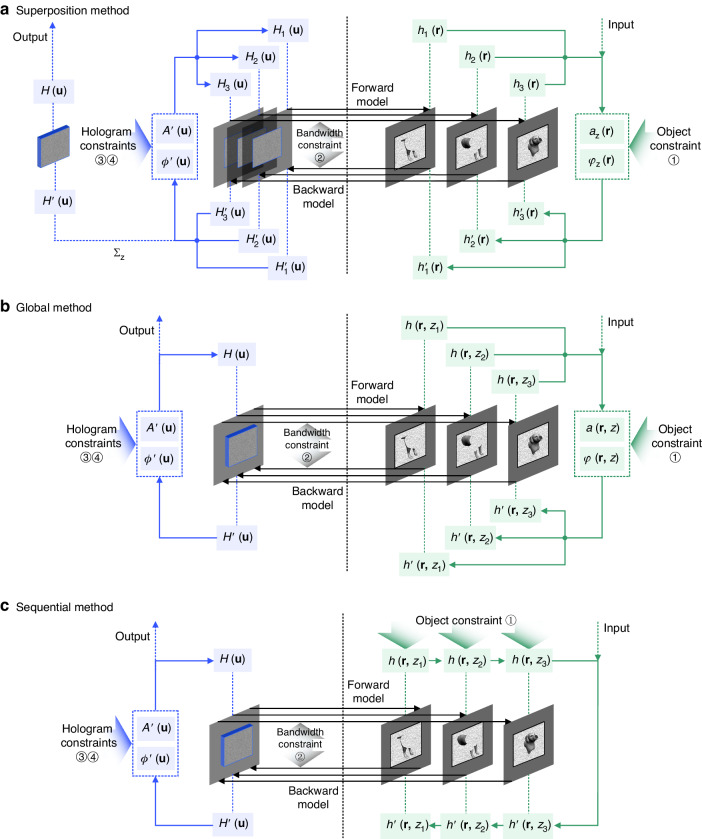
Schemes of alternating projection algorithms to optimize 3D holograms. **a** superposition method. **b** global method. **c** sequential method

Despite the existence of the point-cloud sampling strategy^163^ and the polygon-based sampling strategy^164^, in the optimization pipelines for 3D holograms, the object wave is mainly partitioned by layers^165–167^. One ancient approach to optimization for 3D holography is oriented from wavefront superposition^58,105^, which is a very conventional principle of holography^168^. As is shown in Fig. 7a, the alternating projections occur in each single layer of the object wave ${h}_{l}({\bf{r}})$ with the object intensity constraint ① imposed on each ${h}_{l}({\bf{r}})$. ${z}_{l}$ represents the propagation distance for the ${l}^{{\rm{th}}}$ layer. Each projection generates an optimized diffractive wavefront ${H}_{z}({\bf{u}})$ for every sampled depth *z*. The final hologram is synthesized by the superposition of all the wavefronts.

The superposition method has been applied to the optimization of holograms, which is especially preferred in the synthesis of CHs^169^ and AOMs^170^. This is due to the fact that the superposition itself does not break any constraints on CHs, while the hologram intensity constraint ④ for POHs can be violated after superposition^171–173^. To enable a better generation of POHs with the superposition method, a middle complex-amplitude plane is usually added to receive the superposition of waves from multiple depths^136,174,175^. Superposition optimization enables the preservation of full-depth cues of the object wave and possesses a competitive edge in applications like 3D display^176^. However, it brings about a degradation in reconstructed accuracy for POHs. The optimization part of the superposition method is essentially about solving planer optimization problems, which can be implemented by alternating projections, SGD, and the quasi-Newton method.

After the wide utilization of superposition optimization, the idea of global optimization is developed^177^, which enables a broader range of applications of CGH, especially those based on POHs. As is shown in Fig. 7b, the projections occur between the object volumetric matrices *h*(**r**, *z*) and the hologram plane, with the object intensity constraint ① imposed on the 3D matrix of the object wave *h*(**r**, *z*). Because the hologram intensity constraint ④ restricts the hologram $H^{\prime} ({\bf{u}})$ throughout the optimization, the capability of such a global method in generating appropriate POHs is highlighted. A distinct characteristic of global optimization is that the dark voxels on the front layer are also involved in the optimization, blocking the desired intensity on the back layer. The crosstalk breaking performance becomes more remarkable when an object wave is scattered and reconstructed with speckles, but still requires slow phase changes between consecutive planes. This characteristic makes POHs generated by the global method more applicable in fields like optical encryption^178^, 3D beam shaping^179–181^, and data storage^176,182^. However, the global method is yet less suitable for 3D display^183^, in which the crosstalk breaking disturbs the visibility of distant scenes and results in an unreasonable energy distribution^100^. Intensive modifications have been made to improve the performance of the global methods^184–186^, and a corresponding pre-optimization scheme has also been proposed^187^. The 3D optimization of POHs with the global method can be carried out by alternating projections, SGD, and the quasi-Newton method^68,188^. However, due to the difficulty in selecting appropriate outputs for gradient-descent frameworks, the feasible global optimization of 3D CHs is currently more restricted within algorithms achieved by alternating projections^189^.

Sequential optimization is another viable scheme that can satisfy the hologram intensity constraint ④ for POHs. An earlier form of the sequential method was the optimization between object planes, called the ping-pong algorithm^190^. Then the hologram plane was also included in optimization^191,192^. As is shown in Fig. 7c, the sequential method is characterized by sequential propagations from distant object planes $h^{\prime} ({\bf{r}},{z}_{l+1})$ to less distant object planes $h^{\prime} ({\bf{r}},{z}_{l})$, and from the nearest object plane $h^{\prime} ({\bf{r}},{z}_{1})$ to the hologram plane $H^{\prime} ({\bf{u}})$. The hologram $H^{\prime} ({\bf{u}})$ is subject to the hologram intensity constraint ④. In order to form the loop, the constrained hologram *H*(**u**) is then propagated sequentially through each object plane and to the most distant one, with the object intensity constraint ① imposed on every $h({\bf{r}},{z}_{l})$. Because of the existence of the dark voxels, sequential optimization possesses a crosstalk-breaking characteristic like global optimization, which makes this method suitable for encryption and storage^193^. The specific problem of the sequential method is that its corresponding reconstructions have decreasing accuracy as the light propagates. And because sequential propagations are required, the implementation of the sequential method is mostly achieved by alternating projections.

**Fig. 8:**
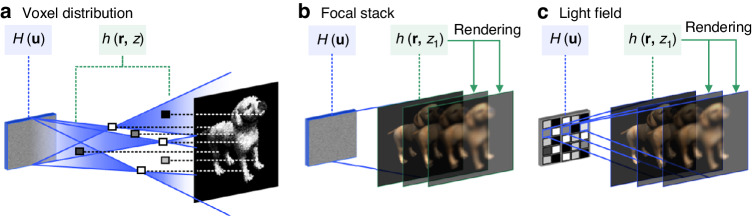
Loss function formulation methods for 3D optimization. **a** voxel distribution method. **b** focal stack method. **c** light field method

Apart from the optimization pipelines, loss function formulation is another crucial factor affecting the performance of 3D holographic reconstruction. In 3D holographic applications, especially for holographic display, not only the accuracy of focus reconstruction is important, but the visual effect of defocus blur is also a great concern in evaluation. The defocused performance of 3D holographic reconstruction is related to the object phase distribution and the visible parallax within the maximum diffraction angle. On this basis, a well-defined loss function can model the spatial variation of diffractive patterns. Representative approaches to cast loss functions include the voxel distribution method, the focal stack method, and the light field method. As is shown in Fig. 8a, the voxel distribution method voxelizes and remaps the object into a 3D volume containing bright voxels and dark voxels^68,69,149^, which is further transferred into the object matrix associated with the loss function. The hologram pixels diffract light to focus on points in space to create bright voxels under illumination. However, it is difficult or even impossible to create a dark voxel in space immediately after a focused bright voxel, because it is physically infeasible to have the phase of light changed rapidly during the free space propagation over a very small distance. For this reason, the reconstruction of the voxel distribution method usually suffers from severe noise and greatly degraded image quality^132^. The focal stack method, as is shown in Fig. 8b, constructs 3D volume by rendering focal stacks at different propagation depths^133,153^. These computationally generated focal stacks at multiple depths are jointly involved in optimization together with the target object, which enables the diffractive reconstruction to better imitate the natural defocus blur, especially in holographic display^194^. The light field method originates from holographic stereograms which reconstruct an array of directional views of the object with a single hologram^139,195–197^, as is shown in Fig. 8c. Although some strategies of light field computation without hogel-based structure have been developed^198^, the application of the light field method is still relatively limited in 3D hologram optimization due to current band limitation existing in the pixelized holographic media like SLMs^199,200^.

**Fig. 9:**
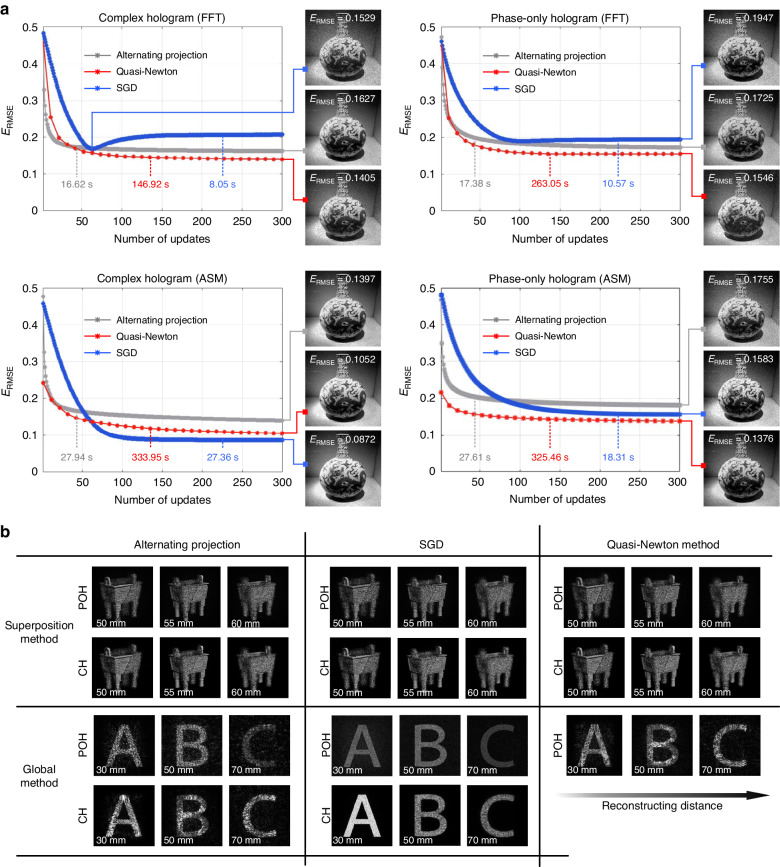
Numerical reconstructions of different optimization frameworks. **a** 2D reconstruction of the optimized complex holograms (CHs) and phase-only holograms (POHs) based on FFT and ASM diffraction model. **b** 3D reconstruction of the optimized complex holograms (CHs) and phase-only holograms (POHs) based on the superposition method and the global method

Here we provide a computational comparison between different optimization frameworks. As presented in Fig. 9a, the optimization of CHs and POHs is demonstrated by basic frameworks of the alternating projections, the SGD method (implemented by the Adam algorithm), and the quasi-Newton method (implemented by the L-BFGS algorithm), respectively. The object phase is initialized with a random matrix. The 512 × 512 holograms are computed on a PC with an Intel Core i9-9900K 3.6 GHz CPU and 32.0 GB of RAM, and an NVIDIA GeForce RTX 2080Ti GPU. Two diffractive propagation methods including the FFT and the ASM are compared, where the propagation distance of ASM is 50 mm. In general, the quasi-Newton method outperforms other optimization frameworks in reconstructing accuracy, which is measured by root-mean-square error (RMSE, ${E}_{{\rm{RMSE}}}=\sqrt{{\sum }_{m,n}{[{a}^{2}({{\bf{r}}}_{m,n})-{I}_{{\rm{obj}}}({{\bf{r}}}_{m,n})]}^{2}}$). However, much more computation time is required for the convergence. Compared with the alternating projections, the holograms optimized by SGD present a slightly lower RMSE and longer time. However, the flexibility in editing the loss functions and the superiority in computing efficiency make SGD unreplaceable in a number of implementations of CGH, especially for the training stage of hologram synthesis based on deep learning methods. The different performances of the first-order and second-order gradient descent methods while approaching convergence are also illustrated. Since SGD is a first-order gradient descent method, biased moment vectors are continuously introduced into the optimization when the algorithm approaches its convergence, resulting in the fluctuation around the optimal solution. On the other hand, the second-order gradient descent method searches for the optimal direction of gradient descent, the output of optimization varies at a limited range around the optimal solution. For this reason, the RMSE curves of SGD optimization in Fig. 9a bounce up when relatively weaker constraints are imposed on the optimization. The optimization convergence is also related to the diffractive calculation approaches utilized. It should be noted that the optimization of FFT-based holograms gives slightly worse quality of reconstruction here. This is because the bandwidth constraint imposed on FFT-based holograms is merged with spatial scale constraint, and thus the searching range of the FFT-based hologram optimization is less restricted around the optimal solution when approaching the convergence. Table 1 provides a review of the performance of these algorithms based on the equal testing of different frameworks.

**Table 1:** Comparison of the numerical performances of different optimization frameworks

		Reconstruction performance	Time	Memory	Loss function	Gradient
Alternating Projection	FFT	Moderate accuracy	Fast	Small	Not required	None
	ASM	Distance-dependent	Fast	Moderate		
SGD	FFT	Unstable convergence	Very fast	Moderate	Required	First-order
	ASM	Distance-dependent	Fast	Moderate		
Quasi-Newton	FFT	High accuracy	Slow	Large	Required	Second-order
	ASM	Distance-dependent	Slow	Large		

The performance of different frameworks for 3D holography is presented in Fig. 9b. Both the superposition method and the global method are demonstrated with the reconstructions of CHs and POHs. Three optimization frameworks perform similarly with 3D holograms generated with the superposition method. As predicted, the diffractive cues for different layers are well preserved after superposition, enabling the generation of defocus dispersion for 3D display. For the global method, however, different optimization frameworks present distinct differences in reconstruction. The quasi-Newton method and SGD are better at breaking the crosstalk between layers and showing more uniform energy distributions, among which SGD performs better at suppressing severe speckle noise. Yet, the multi-depth reconstructions by the alternating projections cannot avoid the disturbance from adjacent layers. This impact is more severe with the sequential method, which is currently only applicable to alternating projection algorithms.

## Initialization

**Fig. 10:**
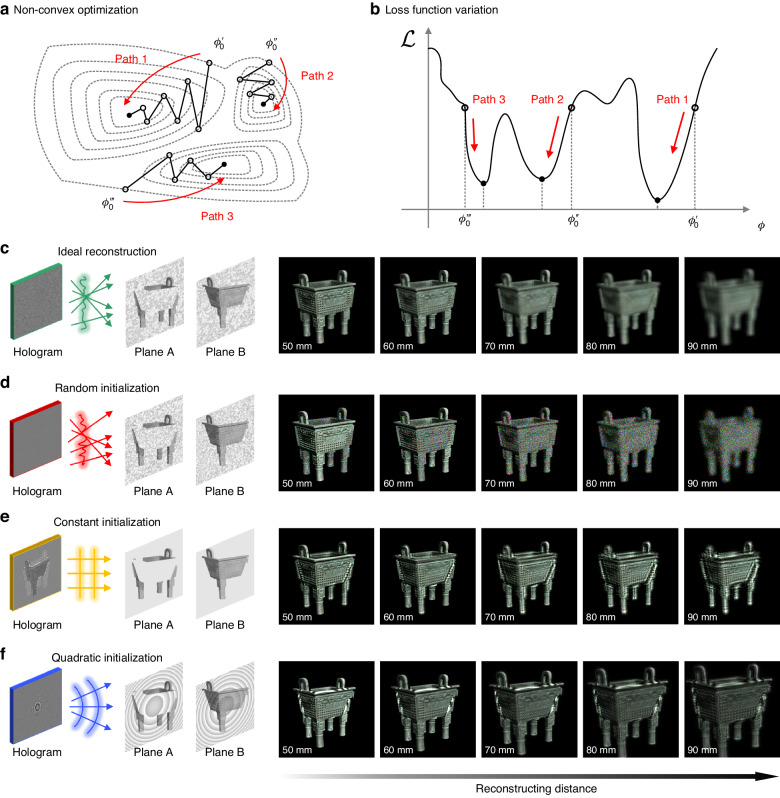
Reconstructions of complex holograms (CHs) at various focal distances when the optimization algorithm is initialized with different object phases. **a** A schematic diagram of the non-convex optimization in an actual hologram synthesis problem. Different optimization paths are triggered by different initialization conditions. **b** The variation of the loss function when a local optimal solution is searched. The closest optimum point is decided by the start point of optimization. **c** A rendered ideal reconstruction for a 3D scene. The reconstructions of CHs optimized with different initialization conditions. **d** Random object phase. **e** Constant object phase. **f** Quadratic object phase

The initialization condition is a crucial factor affecting the optimum solution sought out by an optimization algorithm. Specifically in a hologram synthesis problem, the object phase $\varphi ({\bf{r}})$ is the only free parameter floating in the optimization. Given that the hologram optimization is non-convex, as is depicted in Fig. 10a, the initial value of this free parameter decides at which point the optimization path starts and to which local optimum point the optimization path is heading. Since the closet local optimal solution differs with different initialization conditions, illustrated in Fig. 10b, the choice of the object phase is closely associated with the performance of the in-focus reconstrued intensity. And the initial object phase determines the defocus pattern through the reconstructed phase. Various attempts are made to manipulate the object phase for initialization of the optimization, adapting computer-generated holograms for diverse applications. In this section, different object phase initializations, such as random phase, constant phase, and quadratic phase, and their corresponding reconstructing performances are discussed.

### Random phase

In the experiments of conventional optical holography, the real-world objects that generate the object waves to be recorded have scattering surfaces^201^. This is based on the fact that most materials encountered in the real world are rough on the scale of object wavelength. Various microscopic facets of the rough scattering surfaces of an object contribute randomly distributed phases to the recorded object wave. The random phase is widely used in CGH because the scattering surfaces of real-world objects produce scattered wavelets, which in turn leads to speckles^202^. Although CGH generates holograms through wave computation instead of optical interferometry, the phase term of the object wave needs to be defined according to the phase generated by the real-world object. An artificial object wave computed with a random phase distribution is thus more in line with the real object wave generated from scatterers.

It can be seen that the phase of the object wave is closely related to the defocus effects in reconstruction. Initialized with a random object phase, as shown in Fig. 10d, the reconstructed wave of optimization can generate a natural blur of defocus like that of the scatterers in the real world, which slowly varies on spatial distribution. This capability is essential because reconstructing a 3D scene is the core strength of holography and reproducing the object image faithfully at various positions in space is necessary. From this perspective, random or to some extent random distributions conform better to the phases of waves modulated by real-world objects in most cases. Since scattering materials occupy a considerable proportion in the natural world^203^, preserving the propagating characteristic of a scattered wave and at the same time removing speckles is unavoidable for CGH.

Apart from this consideration, there is an additional reason to use a random phase. In a great number of applications, holograms need to be encoded onto phase-only elements, such as DOEs, metasurfaces, and liquid crystal on silicon (LCoS). The POH operates only on the phase of an incident wave and is generated based on the assumption that a scattered wavefront can be reconstructed with only the phase information. Therefore, using random phase is also an essential procedure to enable the encoding of POHs in specific applications of holography.

However, a significant drawback of introducing a random phase into optimization is the degraded optical reconstruction severely affected by speckle noise. Careful studies have identified that speckles in CGH are closely associated with phase singularities and are regarded as the results of optical vortices^71^. Speckles in a scattered wavefront are found to be intertwined with optical vortices^204^, indicating the existence of phase singularities on which amplitudes are precisely zero and phases are indeterminate. Although scattered wavefronts are artificially generated in CGH, speckles can be suppressed optically when phase singularities are eliminated^205^. Computational approaches to suppress speckles are essentially making modifications to the random phase introduced in the generation of holograms. Optimization algorithms are able to restrict the scattering caused by the random phase to suppress speckles, which is achieved by feeding the object intensity and bandwidth limitation as constraints^67,68^. Some optimizing or training methods, such as camera-in-the-loop training^66,133,142^, can produce more remarkable results by involving optical systems in computation. However, it has been discovered that optimization algorithms are not inherently capable of eliminating all the speckles^205^. Given the connection between the random phase and speckles, intensive research is carried out to replace the random phase with other phase formats, including constant phase, quadratic phase, and some other artificially defined phase distributions, which will be introduced in the following sections.

### Constant phase

Constant phase is increasingly utilized to synthesize holograms due to the invention of double-phase holograms^206^, delivering highly photorealistic reconstructions free from speckles^207,208^. As a choice of initialization, constant phase is more frequently used in 3D hologram optimization with the superposition method, especially for the synthesis of POHs optimized in virtue of middle complex amplitude planes^136^. The constant phase physically describes the phase distribution of extremely smooth surfaces in the natural world, like mirrors or objects with surfaces as smooth as mirrors. Suchlike objects cause less scattering and fewer speckles naturally even when they are illuminated by coherent beams. As is shown in Fig. 10e, when initialized with a constant object phase, the optimized object wave is reconstructed with undiffused diffracting patterns at defocus positions. This kind of defocus effect with diffracting patterns greatly differs from defocus blurs which are generated by scatterers. Constructing object waves with constant phases means replacing the materials of the object with the ones that naturally cannot cause light scattering and produce speckles. Consequently, the object wave would lose the propagating characteristic as a scattered wave and fail to provide a faithful appearance of the object in 3D space. Observed along the direction of propagation, however, constant phase generates diffracting outlines instead of defocus blurs, weakening the depth sensation of 3D scenes.

### Quadratic phase

The quadratic phase enables the production of faithful, speckle-free, planer reconstructions, especially when it is coordinated with alternating projection algorithms. With the improvements enabled by the double freedoms^103,209^ and proper bandwidth limitation^102,210^, alternating projection algorithms are able to overcome ring artifacts brought about by the quadratic phase, which highly promotes the reconstructing performance in holographic projection.

Nevertheless, the intensity pattern attached to the quadratic phase cannot keep its feature size with the propagation of light^172^, which strictly restricts its application within two-dimensional (2D) holographic projections and beam shaping^211^. As is shown in Fig. 10f, the object wave can be constructed by such a quadratic phase for 3D hologram optimization. In the optimization pipeline, the diffractive wavefronts are converted into CHs and reconstructed at various distances. With the convergent quadratic phase, the object wave is reconstructed with undiffused diffracting patterns at multiple defocus positions. And the reconstructed object intensity cannot maintain its feature size with the enlargement of the reconstructing distance. Because of this enlarging characteristic, the application of the reconstruction for the quadratic phase is limited within 2D holographic projection.

Defining the object phase directly as a quadratic phase has a huge advantage in that it can generate holograms efficiently with a single propagation and avoids speckles. And this quadratic phase approach is indeed applicable to 2D projection. However, it has a consequence that the object wave would lose the propagating characteristic as a scattered wave and fail to provide a faithful appearance of the object and maintain the feature size in 3D space. This is why random phase is still widely used in CGH even after such methods were proposed. Another drawback of introducing well-defined phases, like the constant phase and the quadratic phase, is that any perturbation in the optical configuration of reconstruction, including element damage, dust, or varying illumination, can cause severe degradation in the image quality^71^. This property is concluded as the “robustness” brought about by the initial phase and compared in Table 2.

**Table 2:** Comparison of 3D reconstructing performances of different initial phases

	Function	Focus	Defocus	Interlayer transition	Robustness
Random phase	Scatterer	Speckle	Granular blur	Continuous	High
Constant phase	Smoothness	Speckle free	Diffractive pattern	Discontinuous	Moderate
Quadratic phase	Lens and smoothness	Speckle free	Scaled pattern	Discontinuous	Low

### Other phase patterns

Some other attempts at adjusting random phase also include imposing constraints on the phase gradient, controlling the standard deviation of phase radius^212^, and limiting the range of phase radius^213,214^. A regularization term may be added to the objective function to restrict the variation of some parameters, like the gradient of the object phase, in optimization. With these modifications on the object phase, scattering characteristics of the object wave, which is ensured by sufficient randomness and fluctuating ranges, are weakened synchronously with the suppression of speckles, resulting in various distortions in depth dimension^138^. Table 2 provides a general comparison of 3D reconstructing performances of different initial phases.

## Conclusion

In this review article, we have focused on the inverse problem in CGH and provided an overview of various frameworks based on non-convex optimization for appropriate hologram generation. The corresponding algorithms synthesize holograms by searching for optimal solutions within the feasible set restricted by physical models and implementation schemes. Although the computation of holograms is a numerical process, purely computational strategies cannot guarantee the generation of appropriate holograms with faithful reconstruction unless the underlying physics of holography is carefully considered in the calculation. Therefore, this review incorporates an understanding of the fundamental physics in CGH and provides an explanation of the various optimization frameworks, constraints imposed on optimization, 2D/3D optimization pipelines, and object phase initializations utilized in CGH algorithms. Recent advancements in calculation principles and computational strategies have further extended the capabilities of hologram optimization algorithms. By involving cameras or other hardware sets in optimization, the accuracy of planar reconstructions from CGH has been improved significantly. Furthermore, optimization-based hologram synthesis has been enhanced by introducing neural networks, resulting in a significant reduction in computation time to ~1 s^208,215^. Further improvement in the computing pipeline has made the optimization of 3D holograms feasible in practice, resulting in natural-like depth perceptions. These progresses have laid a foundation for the fast advance of CGH and been implemented on a wide range of holographic media, including digital micromirror devices, LCoS, DOEs^119,216^, and metasurfaces^217–220^. Overall, the optimization algorithms have significantly improved the accuracy and efficiency when generating appropriate holograms for faithful reconstruction, while considering the fundamental physics of holography, leading to their deployment in various fields.

## Data Availability

The open-source code for the above-mentioned numerical simulations on 2D and 3D hologram optimization is provided as supplemental materials^221^.
